# Persistent focal pulmonary opacity elucidated by transbronchial cryobiopsy: a case for larger biopsies

**DOI:** 10.1002/rcr2.410

**Published:** 2019-02-27

**Authors:** Jasleen Kaur Pannu, Otis Bryant Rickman, Robert James Lentz, Joyce Evelyn Johnson, Fabien Maldonado

**Affiliations:** ^1^ Division of Pulmonary and Critical Care Medicine Ohio State University Medical Center Columbus OH USA; ^2^ Division of Allergy, Pulmonary and Critical Care Medicine Vanderbilt University Medical Center Nashville TN USA; ^3^ Department of Veterans Affairs Medical Center Nashville TN USA; ^4^ Department of Pathology, Microbiology and Immunology Vanderbilt University Medical Center Nashville TN USA

**Keywords:** *Blastomyces dermatitidis*, blastomycosis, pulmonary infiltrates, pulmonary opacity, transbronchial cryobiopsy

## Abstract

Persistent pulmonary opacities associated with respiratory symptoms that progress despite medical treatment present a diagnostic dilemma for pulmonologists. We describe the case of a 37‐year‐old woman presenting with progressive fatigue, shortness of breath, and weight loss over six months with a progressively worsening right basilar infiltrate on chest imaging in spite of antibacterial therapy. Transbronchial cryobiopsy was used to establish the diagnosis after a bronchoscopy with traditional forceps biopsies was non‐diagnostic. This case demonstrates the value of cryobiopsy as a second‐line strategy for pulmonary infiltrates when aetiology remains unclear after less invasive testing.

## Introduction

Persistent pulmonary opacities that are symptomatic and progress despite medical treatment present a diagnostic dilemma for pulmonologists. Conventional bronchoscopic sampling and biopsy is usually the first step of invasive diagnostic testing. When bronchoscopy is not helpful, surgical lung biopsy is occasionally considered as it provides the best diagnostic yield, but it is rarely pursued due to concerns over morbidity and mortality 1.

Transbronchial cryobiopsy has been the focus of considerable interest in the diagnosis of diffuse parenchymal lung disease 2. Its utility for the diagnosis of persistent focal pulmonary infiltrates has not been explored, and specifically, there is no known utility of cryobiopsy for the diagnosis of pulmonary infection. We report a case of unexplained persistent focal infiltrate after conventional transbronchial forceps biopsy, which was ultimately elucidated by cryobiopsy.

## Case Report

**Figure 1:**
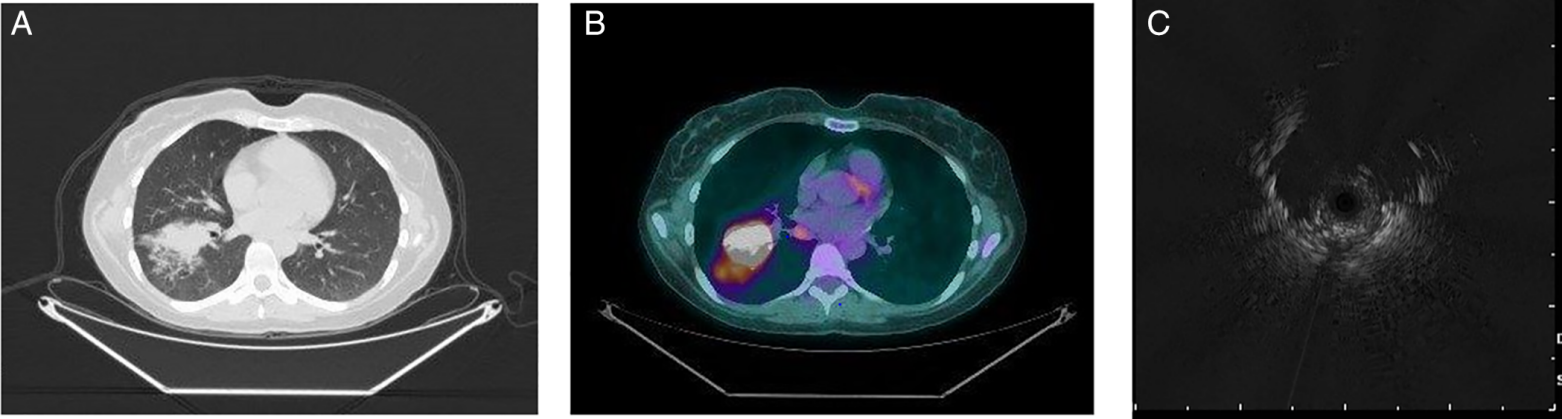
(A, B): Computed tomography (CT) chest showing right lower lobe opacity, 4.4 × 2.9 cm in axial dimension, positron emission tomography avid, and with a mass‐like appearance that was a cluster of nodules and micronodules earlier (17 × 11 mm in largest dimension). There are numerous surrounding micronodules and ground‐glass opacities. (C) Endobronchial radial probe ultrasound image of the lesion.

**Figure 2:**
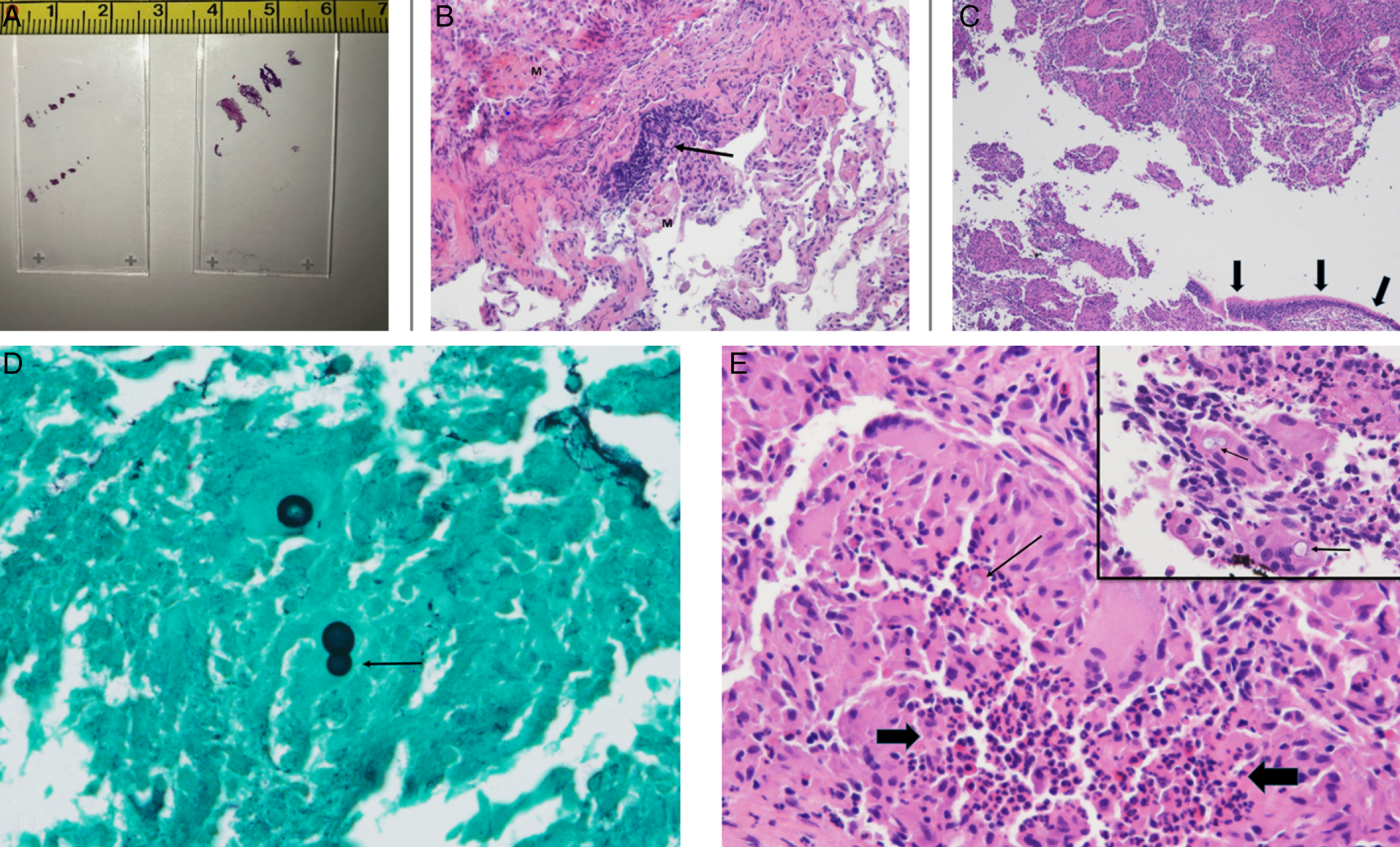
(A) Tissue slides from the two procedures; traditional forceps biopsy on left and cryobiopsy on right. The largest cryobiopsy measures approximately 6–7 mm, versus 2 mm for the traditional forceps biopsy. (B) Medium‐power view of traditional forceps biopsy. Note the increase in alveolar macrophages (M) and focus of hyperplastic bronchus‐associated lymphoid tissue (arrow); no granulomas are present. GMS stain was negative. H and E, 25×. (C) Medium‐power view of the cryobiopsy. One of four biopsies, designated “friable” by the bronchoscopist, shows bronchiolocentric necrotizing granulomatous inflammation; note the intact airway epithelium, lower right (arrows). H and E, 25×. (D) Although blastomyces organisms are often visible on H and E stains, the more sensitive silver stain shows many more fungal yeast forms and highlights details of size and budding. Note the broad‐based budding (arrow). Gomori metheneamine silver, 250×. (E) Close view of a granuloma. Note the central neutrophilic microabscess (between block arrows), characteristic of blastomyces‐associated granulomas. A single organism is identifiable in the large image (fine arrow). H and E, 125×. Inset: closer view of organisms visible on H and E (arrows). H and E, 250×.

A 37‐year‐old Caucasian woman, with a 15 pack‐year ongoing smoking history, presented to our outpatient interventional pulmonary clinic with complaints of progressive fatigue, shortness of breath on exertion, loss of appetite, and a 30‐pound weight loss during the six months preceding presentation. In addition, she also reported nausea with occasional vomiting, dysphagia, and intermittent chest discomfort with lower abdominal pain. Prior diagnostic investigations included chest X‐ray and computed tomography (CT) scans of her chest over the preceding two months, which had demonstrated a persistent and progressively enlarging right lower lobe opacity with clustered nodules and micronodules despite outpatient antibiotic treatment. Bronchoscopy with bronchoalveolar lavage (BAL) and multiple conventional forceps transbronchial biopsies (10 fragments of tissue measuring less than 0.1–0.1 cm in greatest dimension) were suggestive of respiratory bronchiolitis, without malignancy or microorganisms identified (Fig. 2). Histoplasma antigen on the BAL and cultures was negative (bacterial, fungal, acid‐fast bacterial). Positron emission tomography (PET) scan, performed six weeks later due to persistent symptoms, demonstrated substantial interval enlargement of the right lower lobe opacity. It was 4.4 × 2.9 cm in axial dimension, PET avid, and now with a mass‐like appearance that was earlier a cluster of nodules and micronodules (17 × 11 mm in largest dimension). There were numerous surrounding micronodules and ground‐glass opacities (Fig. 1).

In light of this progressive pulmonary process inadequately characterized with traditional bronchoscopic sampling methods, the patient underwent a second bronchoscopy with transbronchial cryobiopsies. Four transbronchial cryobiopsies (0.5 cm in the greatest dimension each) were obtained from different segments targeting peripheral and central areas of the lesion in the involved right lower lobe using a previously detailed technique with a 1.9 mm‐sized cryoprobe without guide sheath (Fig. 2) 2. The biopsy site was first interrogated with radial endobronchial ultrasound probe to confirm appropriate targeting of consolidated parenchyma and presence of significant vasculature. The technique of performing transbronchial cryobiopsies for focal parenchymal lesions is similar to that of diffuse parenchymal lung disease (DPLD), except that the optimal area of biopsy in DPLD is at 1–2 cm from the pleura. Surgical pathology demonstrated necrotizing granulomatous inflammation with neutrophilic microabscesses within the granulomas (Fig. 2). Gomori methenamine silver staining demonstrated frequent large yeast forms with broad‐based budding, consistent with *Blastomyces dermatiditis* (Fig. 2). Magnetic resonance imaging of brain excluded central nervous system involvement. She was referred to the infectious disease service and treated with itraconazole (200 mg twice daily). Since starting treatment her weight has remained stable with decreased cough nausea and fatigue. Follow‐up imaging showed almost complete resolution of right lower lobe consolidation and nodularity.

## Discussion

Pulmonary blastomycosis is an uncommon disease caused by a dimorphic fungal infection endemic in south‐eastern, south central, and mid‐western states of the USA. Pulmonary involvement is reported in approximately 50% of cases 3. More than 80% of the time, non‐invasive samples (sputum, tracheal secretions, gastric wash) are diagnostic 3. When invasive methods are used, bronchoscopy with BAL (67% reported yield) and localized transbronchial biopsies (22% reported yield) are the first approach, followed by more invasive surgical approaches depending on the location of disease 3, 4.

*Blastomyces dermatitidis* has been called “the great masquerader” or “pretender” because of the variety of ways in which it can initially present. While it most commonly causes a dense consolidation resembling bacterial pneumonia, other manifestations include nodule or mass‐like lesions that mimic bronchogenic carcinoma or it may present as acute respiratory distress syndrome (ARDS) 3, 4. Cultures from sputum, BAL, or tissue require up to 30 days for growth with definitive identification and, on average, five weeks between presentation and diagnosis. Misdiagnosis is common, with bacterial pneumonia, malignancy, and tuberculosis being the most common initial considerations. This can lead to additional delays causing significant morbidity in patients owing to several weeks of inadequate antibiotics and dissemination of disease, especially in the immunocompromised.

Few reports have been published regarding the use of diagnostic transbronchial cryobiopsy in non‐interstitial lung disease pathology 5. A case series performed on immunocompromised patients with pulmonary infiltrates reported one case of cryptococcosis diagnosed out of 11 patients with pulmonary infiltrates 5. Other diagnosis included non‐caseating granulomatous inflammation, acute interstitial pneumonitis consistent with drug reaction, non‐specific interstitial pneumonia fibrotic variant, diffuse alveolar damage, and organizing pneumonia 5.

In our patient, transbronchial cryobiopsy diagnosed pulmonary blastomycosis after non‐invasive testing, and bronchoscopy with BAL and forceps biopsy had proven non‐diagnostic. This is, to our knowledge, the first case of blastomycosis diagnosed via bronchoscopic cryobiopsy after negative standard forceps biopsies. Cryo‐freezing has not been associated with a limitation to culture organisms. Transbronchial cryobiopsy is a feasible second‐line strategy before consideration for surgical lung biopsy in undiagnosed pulmonary infiltrates, including infections, if initial non‐invasive testing is not diagnostic.

### Disclosure Statement

Appropriate written informed consent was obtained for publication of this case report and accompanying images.
